# Nasopharyngeal papillary adenocarcinoma harboring a fusion of ROS1 with GOPC: A case report

**DOI:** 10.1097/MD.0000000000024377

**Published:** 2021-01-22

**Authors:** Jinjing Wang, Shuai Luo, Yao Li, Hong Zheng

**Affiliations:** Department of Pathology, the First Affiliated Hospital of Zunyi Medical University, Guizhou, PR China.

**Keywords:** immunohistochemistry, nasopharyngeal papillary adenocarcinoma, next-generation sequencing, ROS1-GOPC fusion

## Abstract

**Rationale:**

Nasopharyngeal papillary adenocarcinoma is a region-specific tumor originating from the nasopharyngeal surface epithelium. Owing to its rarity, more attention has been paid to its clinicopathologic features, while little effort has been made to study the gene abnormalities that drive this tumor. We describe the first case of nasopharyngeal papillary adenocarcinoma harboring a fusion of ROS1 with GOPC.

**Patient concerns:**

A 22-year-old female patient was diagnosed with nasopharyngeal papillary adenocarcinoma in our hospital, and she had right nasal obstruction for more than 6 months. Nasal endoscopy revealed a mass on the posterior roof of the nasopharynx.

**Diagnoses:**

Immunohistochemical staining showed that the tumor cells were diffusely positive for transcription termination factor 1, vimentin, CK19, glypican-3, and CK7, and negative for melanocyte, CK5/6, CK20, P53, P63, S100, smooth muscle actin, p16, PAX8, and thyroglobulin. The Ki-67 index was approximately 5%; EBV-encoded small nuclear RNA was negative.

**Interventions:**

The tumor was completely excised on endoscopy with a negative surgical margin.

**Outcomes:**

No sign of recurrence was observed during the 3-year follow-up period.

**Lessons:**

Owing to its rarity, pathologists should be aware of this unusual neoplasm to avoid misdiagnosis. Further studies are needed to further characterize the relationship between ROS1-GOPC fusion and the pathogenesis of this carcinoma and its response to tyrosine kinase inhibitors in relapsed cases.

## 1 Introduction

Nasopharyngeal papillary adenocarcinoma is a region-specific tumor originating from the nasopharyngeal surface epithelium.^[1]^ It is an exceedingly rare tumor and accounts for less than 0.5% of nasopharyngeal malignant tumor cases. Histologically, nasopharyngeal papillary adenocarcinoma is characterized by papillary fronds and crowded glandular structures that are covered by cuboidal to columnar epithelium, like the cells of papillary thyroid carcinoma (PTC). Because this tumor is exceedingly rare, many studies have focused on its clinicopathologic features, while little effort has been made to study the gene abnormalities that drive this tumor.^[2]^ Herein, we report a case of a young woman diagnosed with nasopharyngeal papillary adenocarcinoma that harbored a fusion of ROS1 with GOPC.

## 2 Case presentation

**Figure 1:**
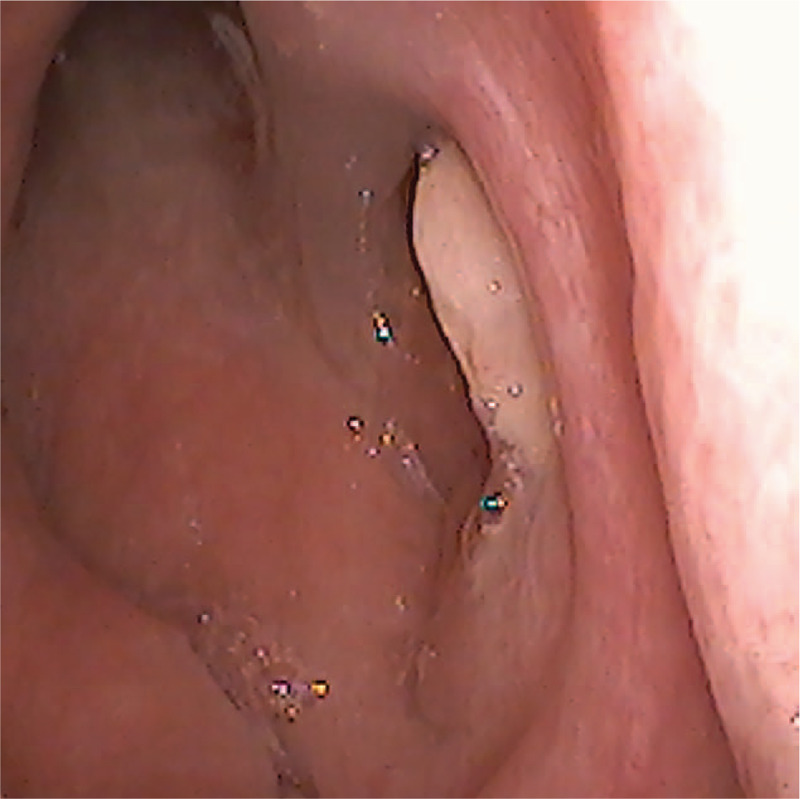
Shape of the nasopharyngeal papillary adenocarcinoma. The mass has a smooth surface, no ulceration, and no bleeding.

**Figure 2:**
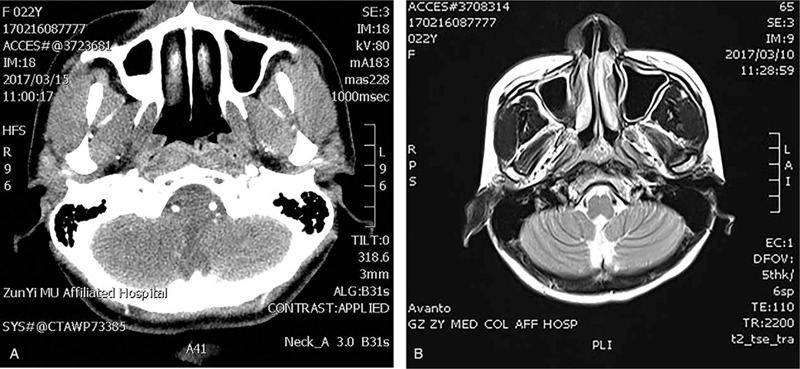
Computed tomography (CT) imaging and enhanced scanning of the head and neck confirmed a soft mass on the posterior of the nasopharynx (A). The tumor exhibited weak contrast enhancement (B).

A 22-year-old woman visited our hospital with complaints of right nasal obstruction for more than 6 months. She occasionally experienced bloody nasal mucus, purulent rhinorrhea, and ear symptoms. No cervical lymph nodes were palpable. No other abnormalities were found during physical examination. Nasal endoscopy found a mass measuring approximately 1 cm in diameter on the posterior roof of the nasopharynx. The mass had a smooth surface, with no ulceration or bleeding (Fig. 1). Computed tomography and contrast-enhanced scanning of the head and neck confirmed a soft mass on the posterior aspect of the nasopharynx, and it exhibited weak contrast enhancement (Fig. 2). The mass did not extend into the sphenoid sinus, clivus, or intracranial space. Computed tomography and ultrasound sonography of the thyroid and chest showed no abnormalities.

**Figure 3:**
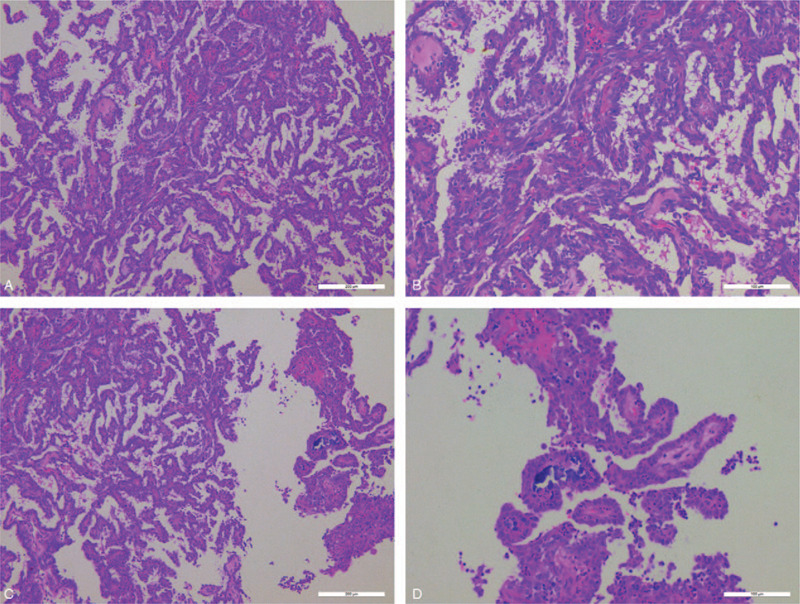
The tumor is composed of complex, arborizing papillae with hyalinized fibrovascular cores (A). Papillae are lined by a single layer of cuboidal to columnar cells (B). the nuclei vary from round to oval and have moderate membrane irregularity with vesicular to clear chromatin. Nuclear pseudo-stratification and overlapping were seen, and scattered nuclei with grooving were present (C). Psammomatoid calcification was found (D).

A biopsy (1.1 cm × 1.2 cm × 1.2 cm) was performed from the mass to clarify its pathological properties. The tumor had a smooth outer surface. Serial sectioning revealed a soft, gelatinous, tan cut surface. Microscopic examination revealed that the tumor was invasive and involved the surface of the epithelium. The tumor was composed of intricate, arborizing papillae with hyalinized fibrovascular cores. The papillae were lined with a single layer of cuboidal to columnar cells with a moderate amount of eosinophilic cytoplasm. Transition from normal epithelial lining cells to tumor cells was identified. The tumor cells exhibited only a minor degree of pleomorphism and hyperchromatism. No mitotic figures or necrotic tissue were found. Similar to those seen in PTC, the nuclei varied from round to oval and had moderate membrane irregularities, with a vesicular to clear chromatin. Nuclear pseudo-stratification and overlapping were seen, and scattered nuclei with grooving were present. Psammomatoid calcifications were found. (Fig. 3).

**Figure 4:**
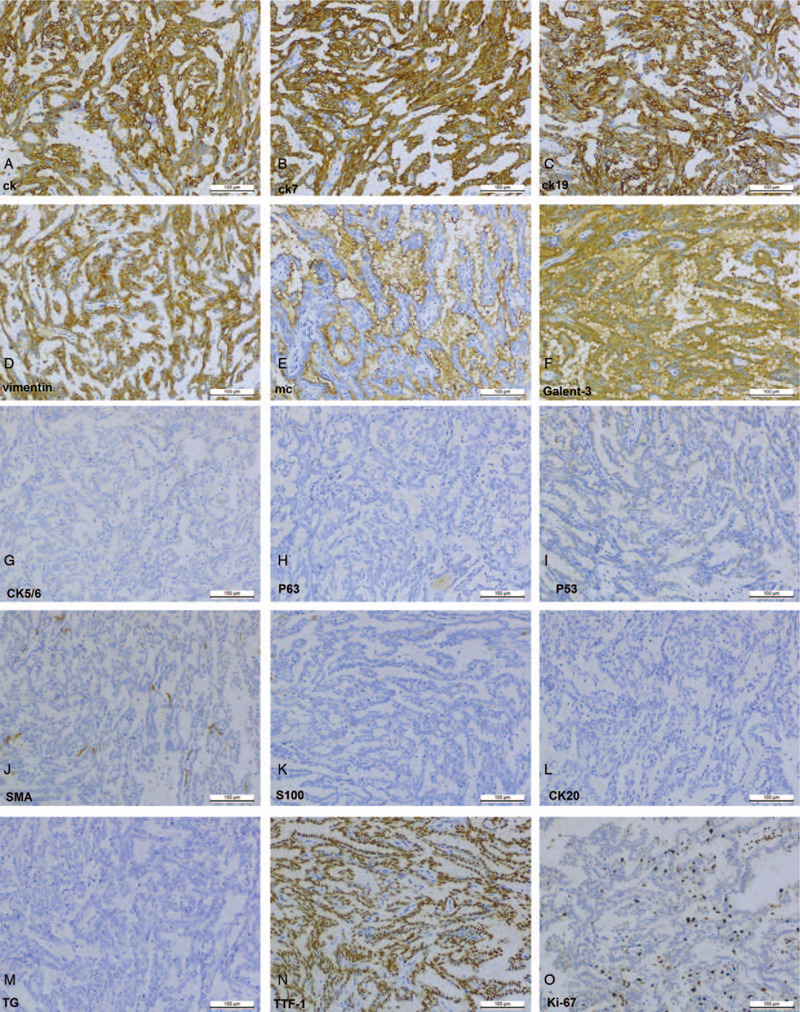
Tumor cells were diffusely positive for CK, CK7, CK19, TTF1, and glypican-3. The cells were negative for MC, P63, SMA, and TG (H&E, ×100).

Because nasopharyngeal papillary adenocarcinoma displayed a striking resemblance to PTC, it was important to exclude nasopharyngeal metastasis from papillary adenocarcinoma of the thyroid gland for an accurate diagnosis. Therefore, an immunohistochemical panel was performed using commercially available antibodies to the following antigens: cytokeratin (CK), CK19, vimentin, transcription termination factor 1 (TTF1), glypian-3, CK7, CK20, CK5/6, melanocyte (MC), P53, P63, S100, smooth muscle actin (SMA), thyroglobulin (TG), Ki-67, CDX-2, P40, glial fibrillary acidic protein (GFAP), and epithelial membrane antigen (EMA). In situ hybridization for the presence of small Epstein-Barr virus (EBV)-encoded RNA was performed to identify the association between the tumor and EBV. All protocols were employed according to the manufacture's recommendations. The tumor cells were diffusely positive for TTF1, vimentin, EMA, CK19, glypican-3, and CK7 (Fig. 4) and were negative for MC, CK5/6, CK20, P53, P63, S100, SMA, and TG. The Ki-67 index was approximately 5%, whereas EBV-encoded small nuclear RNA in situ hybridization was negative. Thus, from the above observations, we identified the tumor as a low-grade malignant thyroid papillary adenocarcinoma of the nasopharynx/nasopharyngeal papillary adenocarcinoma.

The tumor was completely excised on endoscopy with a negative surgical margin. Two days after surgery, the patient was discharged from the hospital. She did not receive adjuvant radiation therapy or chemotherapy. The patient has remained free of local recurrence, and distant metastasis during postoperative follow-up (3.5 years).

To investigate the gene abnormalities that drive this tumor, next-generation sequencing of the biopsy specimen was performed in a clinical pathology laboratory (Anorode, China, Beijing), as previously described. Briefly, ≥50 ng DNA was extracted from a 40-micron tumor sample in formalin-fixed, paraffin-embedded tissue blocks using the phenol-chloroform method. Gene sequencing was performed and targeted all coding exons in 230 cancer-related genes using Illumina HiSeq (Illumina, San Diego, CA) technology. The results of gene sequencing showed a fusion of ROS1 with GOPC. Additional genetic mutations were found in BCR, EGFR, ERBB2, FGFR2, KDR, MET, and TSC2. Mutations in BRAF and RAS were not detected. This is the first report of a fusion of ROS1 with GOPC in a nasopharyngeal papillary adenocarcinoma.

### 2.1 Ethical approval and consent

This case report was approved by the Ethics Committee of the Affiliated Hospital of Zunyi Medical University. Written informed consent was obtained from the patient for publication of this clinical case report.

## 3 Discussion

In the recently published 4th edition of the World Health Organization classification of head and neck tumors, carcinomas of the nasopharynx are categorized into the following three entities: nasopharyngeal carcinomas, papillary adenocarcinomas, and salivary gland tumors.^[3]^ Nasopharyngeal papillary adenocarcinoma is a region-specific tumor originating from the nasopharyngeal surface epithelium and is usually localized in the roof of the nasopharynx and the posterior edge of the nasal septum. It is a low-grade adenocarcinoma found in the nasopharynx with predominately papillary architecture. Wenig et al first described this special adenocarcinoma as a distinct entity and named it as a thyroid-like low-grade nasopharyngeal papillary adenocarcinoma (TL-LGNPPA).^[4]^ Because this adenocarcinoma has low-grade morphology and indolent clinical behavior, they proposed that it should be regarded as a distinct entity from conventional adenocarcinomas in this region. This adenocarcinoma was enrolled in the 2005 World Health Organization classification system of malignant epithelial tumors of the nasopharynx.^[5]^

This tumor can occur in patients of any age, with a reported range from 9 to 64 years. To date, only 42 cases of nasopharyngeal papillary adenocarcinoma have been reported in the literature, including 26 reports in English and 16 in Chinese.^[2,6–8]^ No gender predisposition is reported for this tumor. Histologically, this tumor exhibits papillary architecture with fibrovascular cores lined by cuboidal to columnar epithelium, eosinophilic cytoplasm, and round-to-bland oval nuclei of the epithelial cells, overlapping nuclei with optically clear chromatin, and scattered psammoma bodies. These features are very similar to thyroid papillary adenocarcinoma. In some previously reported cases, the transition from the normal surface respiratory epithelium to tumor cells was identified,^[2,6–8]^ all of which were also observed in the present case.

Because the morphology of nasopharyngeal papillary adenocarcinoma is similar to thyroid papillary adenocarcinoma, an immunohistochemical panel should be used to either determine the subgroup or to exclude the existence of metastatic tumors, especially from the thyroid. The immunostaining analysis showed positive staining for vimentin, CK19, TTF1, glypican-3, and CK7 as well as negative staining for MC, CK5/6, CK20, P53, P63, S100, SMA, and TG, distinguishing the tumor from metastasis of the PTC . In addition to microscopic findings, thyroid imaging findings in our investigation (of the thyroid gland) were normal. Thus, in the above observations, our case was typical of nasopharyngeal papillary adenocarcinoma.

Based on its low grade and its occurrence near the salivary gland tissue, the following differential diagnoses were considered in our case: polymorphous low-grade adenocarcinoma (PLGA) of the salivary gland and acinic cell carcinomas (ACC). PLGA manifests as a papillary structure with a cribriform pattern and solid growth of cuboidal cells; the proximity of the present case to the salivary gland tissue and its P63 negativity was indicative of these features. PLGA is more aggressive and positive for vimentin and the S100 protein. To date, positivity for TTF-1 has never been reported in PLGA. ACCs, with a papillary component, are frequently cystic and variably positive for the S100 protein and vimentin, according to the range of differentiation.

The etiology and pathogenesis of nasopharyngeal papillary adenocarcinoma remain to be elucidated. Studies have consistently revealed EBV to be closely linked with the development and carcinogenesis of nasopharyngeal carcinoma, particularly evidence of the non-keratinizing histology. However, the relationship between nasopharyngeal papillary adenocarcinoma and EBV is still uncertain. We tested EBV-encoded small nuclear RNA in situ hybridization on our patient's tumor and found it to be negative. Our result is similar to the results reported by Wu et al and Fu et al,^[9,10]^ indicating that EBV has no role in the pathogenesis of these tumors.

BRAF and RAS mutations, the most common gene alterations occurring in PTC, were not detected in our case of nasopharyngeal papillary adenocarcinoma. Therefore, despite their histological similarities, the carcinogenesis processes of nasopharyngeal papillary adenocarcinoma and PTC are probably different. Nasopharyngeal papillary adenocarcinoma and PTC do not share the same molecular pathogenesis. Similarly, Oishi et al. reported negative immunohistochemical staining with the BRAFV600E mutant antibody.^[9,11,12]^

The *ROS1* gene is located on the long (q) arm of chromosome 6 at position 22. The *ROS1* gene is highly conserved from drosophila through zebrafish, rat, cow, rhesus, and *Homo sapiens*. ROS1 is a receptor tyrosine kinase of the insulin receptor family and is involved in downstream signaling processes involved in cell growth and differentiation.^[13]^ Chromosomal rearrangements of ROS1 result in constitutionally active kinases that stimulate multiple pathways such as JAK-STAT, PI3K-AKT-mTOR, and RAS-RAF-MEK-ERK.^[3–16]^ Fusion products of ROS1 have been observed in many cancers, including cancers of the lung, gastrointestinal tract, hepatobiliary tree, and the central nervous system.^[13–18]^ ROS1 rearrangements have been documented with the following fusion partners; CCDC6, CD74, CEP85L, GOPC/FIG, and TPM3. ROS1-GOPC/FIG fusion was observed in this patient's tumor. The identification of a ROS1-GOPC fusion has significant clinical implications due to the established anti-cancer activity of tyrosine kinase inhibitors (TKIs) in tumors that carry ROS1 rearrangements.^[19–21]^ The ROS1 tyrosine kinase inhibitor (TKI) crizotinib shows marked clinical efficacy in TKI-naive *ROS1*-positive patients in clinical trials, with an overall response of 65% to 72% and an expected median progression-free survival of 19 months. Crizotinib is recommended in the first-line setting for *ROS1*-positive patients. Lorlatinib is a potent, orally available, CNS-penetrant, selective ROS1 TKI. Lorlatinib is an effective treatment option after crizotinib failure in *ROS1*-positive patients. Many other ROS1 TKIs with promising activity such as entrectinib, brigatinib, repotrectinib, and DS-6051b are being investigated and could extend the survival of *ROS1-*positive patients.^[21]^

According to the literature review, patients with nasopharyngeal papillary adenocarcinoma have an excellent prognosis with almost no recurrence. Surgical resection is currently the mainstay of treatment for the primary nasopharyngeal papillary adenocarcinoma, but some patients also received radiation or chemotherapy. Photodynamic therapy with topical 5-aminolevulinic acid as a postoperative adjuvant therapy for an incompletely resected primary nasopharyngeal papillary adenocarcinoma has been reported.^[22]^ Adjuvant radiotherapy had been suggested for incompletely resected tumors, and 1 patient had accepted postoperative radiotherapy for nasal cavity with a total dose of 60 Gy in 30 fractions over 6 weeks. There was no evidence of recurrence after 4 months of surgery, and further follow-up is being carried out.^[23]^ However, the role of adjuvant radiotherapy is not established as these well-differentiated tumors respond poorly to radiation.^[6]^ In our case, because the surgical margin was negative, and the patient was young, he did not receive adjuvant radiation therapy or chemotherapy. The patient has remained free of local recurrence and distant metastasis during the 3.5-year postoperative follow-up.

Although there are no relapse cases reported, it may be due to short follow-up times. Long-term monitoring should be performed for any future recurrence, should they occur. In case of some patients who had relapse of this tumor, TKIs such as crizotinib and lorlatinib may be considered as treatment options and could bring benefit to the patient.^[2,24–25]^

In conclusion, nasopharyngeal papillary adenocarcinoma is an extremely rare entity, the awareness of which is necessary among pathologists to be aware of this unusual neoplasm to avoid misdiagnosis, as one of its more common morphological mimickers. We describe the first case of nasopharyngeal papillary adenocarcinoma harboring a fusion of ROS1 with GOPC. Further studies are needed to better characterize the relationship between ROS1-GOPC fusion with the pathogenesis of nasopharyngeal papillary adenocarcinoma and response to TKIs such as crizotinib and lorlatinib toward this carcinoma.

## Acknowledgments

The authors would like to thank Editage (editage.com) for English language editing.

## Author contributions

**Conceptualization:** Jinjing Wang, Hong Zheng.

**Investigation:** Shuai Luo, Yao Li.

**Project administration:** Jinjing Wang.

**Resources:** Shuai Luo, Yao Li.

**Writing – original draft:** Jinjing Wang, Hong Zheng.

**Writing – review & editing:** Jinjing Wang, Hong Zheng.
